# Combined regulation of pro-inflammatory cytokines production by STAT3 and STAT5 in a model of *B. pertussis* infection of alveolar macrophages

**DOI:** 10.3389/fimmu.2023.1254276

**Published:** 2023-09-28

**Authors:** Fethi Khiter, Zoulika Kherrouche, Violaine Dubois, Stéphanie Slupek, Emmanuelle Petit, Anne-Sophie Debrie, Stéphane Cauchi, Nicolas Barois, Carine Rouanet, Nathalie Mielcarek

**Affiliations:** ^1^ University of Lille, CNRS, Inserm, CHU Lille, Institut Pasteur Lille, U1019-UMR 9017-CIIL-Center for Infection and Immunity of Lille, Lille, France; ^2^ Univ. Lille, CNRS, Inserm, CHU Lille, Institut Pasteur de Lille, UMR9020-U1277-CANTHER-Cancer Heterogeneity Plasticity and Resistance to Therapies, Lille, France

**Keywords:** *Bordetella pertussis*, alveolar macrophages, infection, cytokines, STAT proteins

## Abstract

*Bordetella pertussis* is a highly contagious respiratory pathogen responsible for whooping-cough or pertussis. Despite high vaccination coverage worldwide, this gram-negative bacterium continues to spread among the population. *B. pertussis* is transmitted by aerosol droplets from an infected individual to a new host and will colonize its upper respiratory tract. Alveolar macrophages (AMs) are effector cells of the innate immune system that phagocytose *B. pertussis* and secrete both pro-inflammatory and antimicrobial mediators in the lungs. However, understanding their role in *B. pertussis* pathogenesis at the molecular level is hampered by the limited number of primary AMs that can be collected *in vivo*. In order to decipher the regulation of innate response induced by *B. pertussis* infection, we used for the first time self-renewing, non-transformed cells, called Max Planck Institute (MPI) cells, which are phenotypically and functionally very close to pulmonary AMs. Using optimized infection conditions, we characterized the entry and the clearance of *B. pertussis* within MPI macrophages. We showed that under these conditions, MPI cells exhibit a pro-inflammatory phenotype with the production of TNF, IL-1β, IL-6 and MIP-2α, similarly to primary AMs purified from broncho-alveolar fluids of mice. In addition, we explored the yet uncharacterized role of the signal transduction activator of transcription (STAT) proteins family in the innate immune response to *B. pertussis* infection and showed for the first time the parallel regulation of pro-inflammatory cytokines by STAT3 and STAT5 in MPI macrophages infected by *B. pertussis*. Altogether, this work highlights the interest of using MPI cells for experiments optimization and preliminary data acquisition to understand *B. pertussis* interaction with AMs, and thus significantly reduce the number of animals to be sacrificed.

## Introduction

Whooping cough or pertussis is a highly transmissible respiratory disease, responsible for more than 19 million cases worldwide and 117,000 deaths in children under 5 years old reported in 2019 (1). Despite being a vaccine-preventable disease, whooping cough still represents a major health problem due to an increasing incidence which has been observed since the early 2010s (2). The inability of current acellular pertussis vaccines to prevent nasal infection and transmission and to induce long-lasting protection are the main reasons for the resurgence of pertussis (3). Whooping cough is caused by the human-restricted pathogen *Bordetella pertussis*, a gram-negative bacterium transmitted by aerosol droplets (4). Once inhaled from an infected person, *B. pertussis* will express adhesins and toxins which will promote the colonization of the respiratory tract of the newly infected host and will induce damages in the first line of defense which is the airway epithelium. Resident and recruited innate immune cells such as alveolar macrophages and dendritic cells will be among the first immune cells to sense and to respond to *B. pertussis* infection in the lungs (5).

Alveolar macrophages (AMs) are the major macrophage population in the lungs (6). Their role during *B. pertussis* pathogenesis is twofold. These professional phagocytic cells play an important role in controlling infection, since AM depletion in mice promotes *B. pertussis* infection (7). This mechanism involves a Myd88-Adaptator-Like protein (MAL) dependent-pathway and the early induction of pro-inflammatory cytokines and chemokines (8). As well as contributing to protective immunity, AMs may also provide a niche for prolonged *B. pertussis* survival. Indeed, *B. pertussis* have been found inside AMs of infected infants and children with severe pertussis (9, 10). Similarly, intracellular survival was observed in mouse models (7, 11) as well as *in vitro* using human and mouse macrophages (8, 12).

Macrophages are a heterogeneous cell population which differ phenotypically and functionally depending on their origin and environment (13). AMs derived from embryonic progenitor and their development and self-renewal capacity depends on granulocyte colony-stimulating factor (GM-CSF) produced by type II alveolar epithelial cells (14, 15). Nevertheless, most of the current knowledge on macrophage responses to *B. pertussis* infection were extrapolated from data obtained using cells derived from human mononuclear cells (16), monocytic cell lines such as the THP1 (17, 18), or bone-marrow derived macrophages (8). Indeed, studying interactions between pathogens and alveolar macrophages is hampered by the limited number of AMs which can be obtained from naïve mice. In the present study, we used the recently described Max Planck Institute (MPI) cells to study the alveolar macrophage response to *B. pertussis* infection. MPI cells are non-transformed, GM-CSF-dependent murine macrophage cell line established from fetal liver cells which present phenotypical and immunological similarities with primary murine alveolar macrophages, such as the CD11c expression, low levels of CD14 and the pattern of pro-inflammatory cytokines production that differs from those of bone-marrow derived macrophages (19). Those cells have been shown to be a valuable model to study the host-pathogen interaction, such as with *P. aeruginosa* (20), *M. tuberculosis* (21), and more recently with *S. pneumoniae* (22). Here we set up an optimized protocol for infection of MPI cells by *B. pertussis* and characterized the process of bacterial internalization and clearance. We compared the production of pro-inflammatory cytokines and chemokines by infected MPI cells and primary AMs isolated from broncho-alveolar lavage fluids of mice. Using the valuable cellular model that are MPI cells, we explored the mechanism regulating the expression of pro-inflammatory cytokines by *B. pertussis* and the involvement of the family of STAT (signal transduction activator of transcription) proteins. Our results showed that the combined inhibition of STAT3 and STAT5 is associated with a high level of pro-inflammatory cytokine production by MPI macrophages in response to *B. pertussis* infection.

## Materials and methods

### *Bordetella pertussis* strain and growth conditions

Gentamicin-resistant derivative of *B. pertussis* B1917 bacteria (23, 24) were grown for 36h at 37°C on Bordet-Gengou (BG) agar (Difco) supplemented with 1% glycerol, 10% of Sheep Defibrinated Blood and 10 µg/ml gentamicin (Sigma-Aldrich). The bacteria were then scrapped off the plates and suspended in RPMI medium at the desired optical density.

### MPI model

Max Plank Institute (MPI) macrophages are self-renewing, non-transformed, GM-CSF dependent murine cells, immunologically and phenotypically similar to murine alveolar macrophages (19). MPI were routinely cultivated in non-treated T-75 flasks (Sarstedt) at 37°C, 5% CO_2_ in RPMI 1640-Glutamax medium (Gibco), supplemented with 10% Fœtal Bovine Serum (Eurobio) and 30 ng/ml GM-CSF (Miltenyi). All experiments were performed using MPI cells from less than 40 passages.

For infection experiment, adherent cells were gently detached with Versene (Gibco) for 5 minutes at 4°C, recovered by centrifugation. and then seeded in 6 or 96-well plates in RPMI1640-Glutamax medium + 10% FBS, complemented or not with 15 ng/ml GM-CSF.

### Isolation of primary murine alveolar macrophages

8-week old C57BL/6 mice were purchased from Charles River, Laboratories, France. All mice were maintained at the Institut Pasteur de Lille BSL-2 animal facility. Animal experiments were carried out according to the guidelines of the French Ministry of Research on animal experiments and with institutional regulations and ethical guidelines (B59-350009, Institut Pasteur de Lille, France). The protocols were approved by the Ethical Committees of the Region Nord-Pas-de-Calais and the Ministry of Research (agreement number APAFIS # 201603311654342_v2). To recover broncho-alveolar lavage fluids (BALFs), mice were euthanized by intraperitoneal injection of pentobarbital (Euthasol, TVM Lab). A canule was inserted in the trachea and BALFs were recovered by lavages with 800 µl of a pre-warmed solution of PBS (Gibco), 2% FBS and 2 mM EDTA and centrifuged at 300g, at 4°C for 5 min. Erythrocytes were lysed using ACK Lysing Buffer (Gibco). Cell pellets were resuspended in RPMI-1640-Glutamax, 10% FBS, 1 mM Pyruvate (Gibco) and GM-CSF (15 ng/ml) and were seeded in 96-well tissue-culture treated plates (CytoOne) at a final concentration of 2 x 10^5^ cells/well. Purity of alveolar macrophages was assessed by Facs analysis and was shown to be higher than 90% (**Figure S1**).

### Macrophages infection with *B. pertussis*

Macrophages were seeded in 6 or 96-well plates (CytoOne) at a concentration of 2 x 10^5^ cells/well, with or without GM-CSF (15 ng/ml) and incubated at 37°C under 5% CO_2_. After 24h of incubation, the macrophages were infected with *B. pertussis* for 30 min, 1h or 2h at a multiplicity of infection (MOI) of 10, 50 or 100 bacteria/cell. The end of the contact time corresponds to T0. Non-adherent *B. pertussis* were removed by two successive washes with PBS and remaining extracellular bacteria were killed by 1h treatment with 100µg/ml polymyxin B (Euromedex) diluted in the culture medium. Cellular activity was assessed at different time points after infection using the MTT assay (Cell Kit Proliferation I, Roche) and measurement of the Optical Density (OD) on a plate reader at λ = 570nM. The results are expressed as the ratio of the (OD_stimulated cells_/OD_unstimulated cells_) *100. To evaluate the intracellular bacterial loads, macrophages were lysed at indicated times with 0,1% saponin (Sigma) and serial dilutions of the lysates were plated on BG agar medium. After 5 days of incubation at 37°C, the Colony Forming Units (CFU) were counted on the plates. Intracellular survival is calculated with the ratio of (CFU_Time of interest_/CFU_T0_)*100. In the experiments aiming to assess the impact of phagocytosis, MPI cells were pre-treated with 10µM of Cytochalasin D (Sigma-Aldrich) 30 min prior to *B. pertussis* infection. After 1h of infection, two washes were performed with PBS containing 100µg/ml polymyxin B prior to cells lysis.

### Transmission electron microscopy

After 2h and 4h of contact with *B. pertussis*, MPI cells were fixed with 1% glutaraldehyde in 0.1 M sodium cacodylate pH 6.8 buffer, at 4°C overnight. Infected macrophages were post-fixed with 1% osmium tetroxide and 1.5% potassium ferricyanide then with 1% uranyl acetate, both in distilled water at room temperature in the dark, for 1h. After washing, samples were dehydrated with increasing ethanol-concentration solutions. Samples were finally infiltrated with epoxy resin and cured at 60°C for 24h. Sections of 70-80 nm thickness deposited on formvar-coated grids were observed at 80 kV with a Hitachi H7500 TEM (Milexia), and images were acquired with a 1 Mpixel digital camera from AMT (Milexia).

### Immunoblot

MPI cells were lysed in 0.1% Triton X-100 (Sigma), 5mM EDTA, 50 mM NaCl, 20 mM Tris-HCl pH 7,4 and 1% of HALT protease/phosphatase inhibitor (Thermofisher) and the suspension was clarified by centrifugation. Protein concentration was determined using Pierce BCA Protein Assay Kit (Thermoscientific). Samples were boiled at 95°C for 10 min in Laemmli buffer containing 1M of dithiothreitol and proteins were separated on 4-15% Mini-protean TGX Gel (Bio-rad). Proteins were transferred to nitrocellulose membranes (Thermoscientific) which were blocked in PBS-Tween 0,1% containing 5% of non-fat milk for 1h and incubated overnight at 4°C with the following primary antibodies from Cell Signaling Technology diluted in the blocking solution: anti-β-actin (clone 8H10D10, dilution 1:1000), anti-pSTAT3 Tyr705 (clone 3E2, dilution 1:1000), anti-pSTAT3 Ser727 (ref 9134, dilution 1:1000), anti-pSTAT5 (clone C71E5 dilution 1:1000) anti-STAT3 (clone 124H6, dilution 1:1000) and anti-STAT5 (clone D2O6Y, dilution 1:1000). The membrane was then incubated with the appropriate HRP-conjugated secondary antibodies for 1h30 at room temperature. Revelation was done using SuperSignal West Femto Maximum Sensitive Substrate kit (Thermofisher) and images were acquired on Amersham Imager 600. Band intensity was quantified using ImageJ software and normalized to loading control β-actin. Relative expression for the condition of interest was then normalized to the one obtained for GM-CSF non-infected cells, which give the fold-protein expression.

### Flow cytometry

To assess the rate of macrophages infected with *B. pertussis*, 10^9^ bacteria were resuspended in 1 ml PBS containing 5 mM EDTA and incubated with 15µg/ml carboxyfluorescein diacetate succinimidyl ester (CFSE) (BD Horizon) at 37°C under agitation for 20 min. The CFSE fluorescence intensity was measured by Fluorescence-activated cell sorting (FACS) on Attune NxT (Thermofischer Scientific) to quantify the percentage of MPI infected cell. The bacteria were then diluted to the desired concentration prior to the infection.

For MPI cells phenotyping, cells were washed in PBS containing 3% FBS and 1 mg/ml sodium azide (Sigma). Cells were then incubated with Fc Block (BD Pharmingen, dilution 1:200), followed by staining with anti-SiglecF APC (Biolegend, clone S1700L dilution 1:400), anti-CD11c BV711 (Biolegend, clone N418 dilution 1:400), anti-CD11b BV605 (BD Horizon, clone M1/70 dilution:1:400), anti-CD16/CD32 FITC (BD Pharmingen, clone 2.4 G2 dilution 1:200), anti-Ly6G PE-CF594 (BD Horizon, clone 1A8 dilution 1:400) and anti-F4/80 PE (eBioscience, clone BM8 dilution 1:300). Cells were washed and fixed in Foxp3/Transcription Factor Staining Buffer Set (eBioscience). FACS samples were acquired on an Attune NxT using the Attune NxT Software (ThermoFisher scientific) and analyzed using the FlowJo v10 Software.

For cell cycle analysis, macrophages were harvested and fixed overnight in 70% ethanol at -20 °C. Cells were then washed in PBS and stained with 10 μg/mL propidium iodide (Sigma) containing RNase A (200 μg/mL; ThermoScientific) at 37 °C for 15 min in the dark. Cellular DNA content was measured from at least 10000 events per sample by LSR Fortessa (Beckman Coulter) and cell cycle distribution was analyzed with FlowJo v10 software with the Dean-Jett-Fox univariate model.

### Cytokine analysis

To determine cytokine secretion level, cell culture supernatants were collected 24h after infection and analysed by enzyme-linked immunosorbent assay (ELISA) and Luminex technology according to the manufacturer’s instructions. The following cytokines were assessed by ELISA: TNF (Mouse TNF ELISA Set II, BD OptEIA), IL-6 (Mouse IL-6 ELISA Set, BD OptEIA), IL-1β (Mouse IL-1β Uncoated ELISA Kit, Invitrogen) and MIP-2α (DuoSet Mouse CXCL2/MIP-2, R&D Systems). When indicated, the production of cytokines was measured by Luminex technology using the following Milliplex Mouse magnetic bead panel (Merck, Millipore): TNF, IL-6, IL-1β, MIP-2α, IL12p40, IL-5, IL-10 and IL-22.

To measure the level of gene expression, total RNA was extracted 6h after *B. pertussis* infection and purified using the NucleoSpin kit (Macherey-Nagel). RNA quantification and RIN number were determined respectively with Nanodrop 2000c (Thermofisher) and the Bioanalyzer 2100 (Agilent). The RNAs were selected with a RIN number > 8. Reverse Transcription was then performed on 300 ng of purified RNA with the Verso cDNA Synthesis kit (Thermo Scientific). The obtained cDNA was mixed with the No ROX SYBR 2X MasterMix blue dTTP (Eurogentec-Takyo) and with specific primers for the genes of interest (GOI) (**Supplementary Table 1**) obtained from Eurogentec. qPCR was then realized with a LightCycler480. The Ct of all GOIs were substracted to the Ct of the macrophage housekeeping gene, Syntaxin-5 gene (*stx5a*) (Tanaka et al, 2017). The 2^-ΔΔCt^ formula was then applied and considered as the fold-gene expression relative to the control condition.

### Statistical analysis

Multiple independent groups were analyzed using first a Kruskal-Wallis test then a Conover *post-hoc* test. The Mann-Whitney U test was used to compare differences between two independent groups. If the p-value was 0.05 or less, the results were considered statistically significant. All the graphs, calculations and statistical analyses were performed by using GraphPad Prism software 141 version 9.3.1 and R software version 4.1.1.

## Results

### Optimization of *B. pertussis* infection in the MPI macrophage model

**Figure 1:**
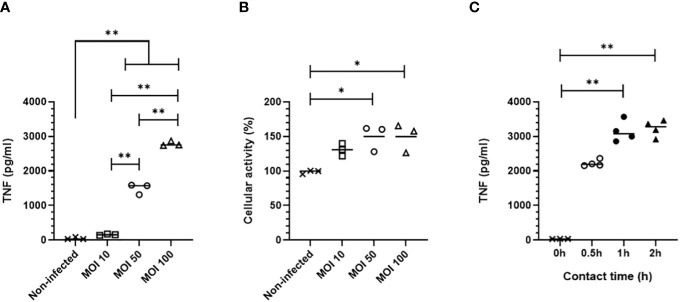
TNF production by MPI cells infected with *B. pertussis*. **(A)** TNF production 24h after infection measured by ELISA from cell culture supernatant of MPI cells incubated for 0,5h with different MOI of *B. pertussis.* **(B)** MPI cell activity assessed using MTT assay, 24h after infection of MPI cells with *B. pertussis* for 0,5h. **(C)** TNF production 24h post-infection measured by ELISA from cell culture supernatant of MPI cells incubated with *B. pertussis* for 0.5, 1 or 2h with a MOI of 50. Lines represent the median of triplicates or quadruplicates. Statistical test used: Kruskal-Wallis followed by a Conover’s test for multiple comparisons. Data are representatives of two independent experiments. *: p-value <0,05, **: p-value <0,01.

We first assessed MPI cell response to *B. pertussis* contact using as readout the secretion of TNF, a pro-inflammatory cytokine produced by *B. pertussis*-infected alveolar macrophages (8). MPI cells were cultured for 30 min with B1917, a virulent strain representative of current circulating *B. pertussis* bacteria (23). We compared increasing doses of B1917 corresponding to 10, 50 and 100 bacteria/cell. We showed that a multiplicity of infection (MOI) of 50 is required to induce significant secretion of TNF by MPI macrophages, and that the level of secreted TNF is increased at a MOI of 100 (**Figure 1A**). In parallel, we measured the impact of *B. pertussis* on MPI activity. Using the MTT assay, we observed a MOI-dependent increased cellular metabolic activity, indicating that B1917 stimulates MPI proliferation (**Figure 1B**). No significant difference was detected between a MOI of 50 and 100 suggesting that the maximum cellular activity level was already reached with the MOI of 50.

*B. pertussis* expresses several adhesins which will interact with receptors at the surface of macrophages to allow bacterial adherence (25–28). We determined the optimal contact time between *B. pertussis* and MPI macrophages to induce a significant TNF response. MPI cells were in contact with B1917 at MOI 50 for 30 min, 1h, or 2h before killing extracellular bacteria with polymyxin B. One day after infection, significant secretion of TNF was measured in cell culture supernatants in all conditions with a peak response induced after 1h of bacterium-cell contact (**Figure 1C**). MPI activity was not affected even after the longest contact time duration with *B. pertussis* (data not shown).

Taken together, these data indicate that the optimized protocol of infection of MPI macrophages with *B. pertussis* strain B1917 requires a contact time of 1h at a MOI of 50. This optimized protocol is used in all the following experiments described in this article.

### Internalization and clearance of *B. pertussis* by MPI macrophages

**Figure 2:**
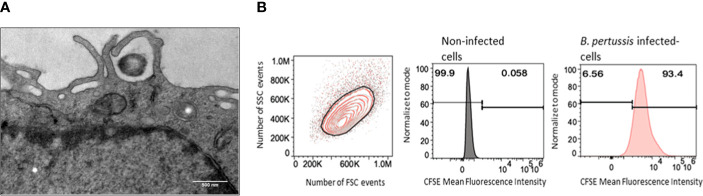
Internalization of *B. pertussis* by the MPI cells. **(A)** Electron micrograph of a cross-sectionned MPI cell incubated for 2h with *B. pertussis* at a MOI of 50. *B. pertussis* is entrapped by cytoplasmic protrusions synthesized by the cell. Scale bar, 500 nm. **(B)** Representative Flow cytometry chart of MPI cells infected for 1h with CFSE-stained-*B. pertussis* at a MOI of 50.

Using transmission electron microscopy, we observed that interaction of *B. pertussis* with MPI induces cellular pseudopods extension which surround bacteria, leading to internalization of *B. pertussis* inside macrophages (**Figure 2A**). We measured the internalization efficiency of *B. pertussis* by MPI using the optimal conditions determined previously. Quantitative assessment by FACS analysis showed that in these conditions, more than 90% of MPI were infected with *B. pertussis* (**Figure 2B**).

**Figure 3:**
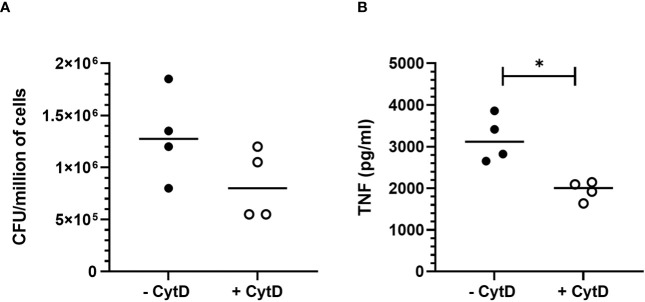
Impact of *B. pertussis* internalization on TNF response by MPI cells. **(A)** Number of colony-forming unit (CFU) internalized by MPI cells pre-treated or not with cytochalasin D (CytD) and incubated with *B. pertussis* for 1h at a MOI of 50. **(B)** TNF production 24h after *B. pertussis* infection measured by ELISA from cell culture supernatant of MPI cells pre-treated or not with Cyt D and incubated with *B. pertussis* for 1h with a MOI of 50. Lines represent the median of quadruplicates. Statistical test used: Mann-Whitney. Data are representatives of 2 experiments. *: p-value < 0,05.

To assess the impact of *B. pertussis* internalization on our readout which is TNF secretion, we treated MPI with cytochalasin D, an inhibitor of actin polymerization and phagocytosis, prior to infection. In these experimental conditions, a non-significant but reproducible reduction in bacterial internalization rate of approximately 40% was observed in MPI treated with cytochalasin D leading to a significant decrease in TNF secretion after infection (**Figures 3A, B**). MPI metabolic activity was not affected by cytochalasin D treatment (data not shown). These data therefore suggest that internalization of *B. pertussis* increases the innate immune responses of infected MPI macrophages compared to stimulation by adherent bacteria alone.

**Figure 4:**
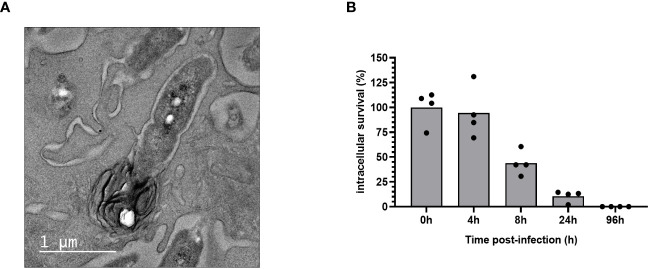
Intracellular clearance of *B. pertussis* by MPI cells. **(A)** Electron micrograph of a cross-sectioned MPI cells after 4h of incubation with *B. pertussis* at a MOI of 50, showing the fusion of a *B. pertussis*-containing phagosome with a lysosome. Scale bar, 500 nm. **(B)** Kinetics of intracellular survival of *B. pertussis* inside MPI cells after 1h of contact between cells and bacteria at a MOI of 50. The end of contact time corresponds to T0. Colony Forming Units of *B. pertussis* from infected-macrophages lysates were counted after 5 days of culture on BG agar plates. The percentage of intracellular survival was calculated with the ratio of (CFU_Time of interest_/CFU_T0_)*100. The bars represent the mean of quadruplicates. Data are representatives of 3 independent experiments.

We observed by transmission electron microscopy that following internalization, *B. pertussis*-containing phagosome fuses with lysosome to form a phagolysosome (**Figure 4A**). We monitored *B. pertussis* intracellular survival inside MPI cells and showed that *B. pertussis* is rapidly cleared by MPI macrophages, with less than 10% live bacteria recovered 24 hours after phagocytosis (**Figure 4B**). Only few live bacteria were recovered 96 hours after infection (<10 CFU/million of MPI cells).

### MPI cells unlike primary alveolar macrophages express high level of pro-inflammatory cytokines but no IL-22 in response to *B. pertussis* infection

**Figure 5:**
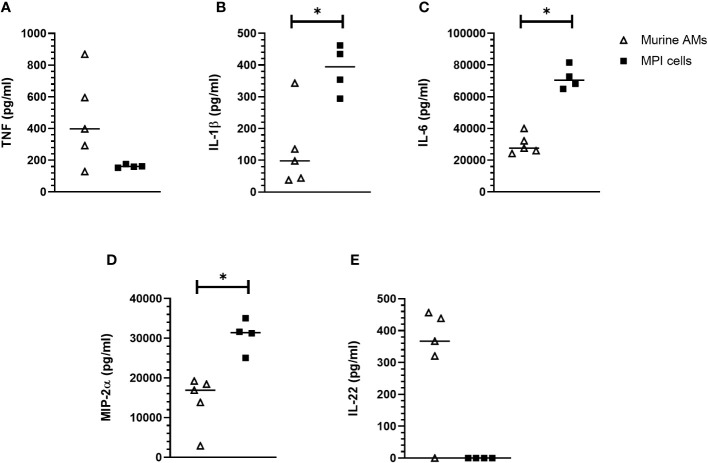
Cytokine responses of MPI cells and primary murine alveolar macrophages infected with *B. pertussis.* MPI cells and AM were incubated for 1h with *B. pertussis* at a MOI of 50. 24h post-infection, production of TNF **(A)**, IL-1β **(B)**, IL-6 **(C)**, MIP-2α **(D)** and IL-22 **(E)** was measured by the Luminex technology. Lines represent the median of five biological replicates and the graph is representative of 2 experiments. Statistical test used: Mann-Whitney. *: p-value < 0,05.

We compared after infection with *B. pertussis¸* the levels of cytokines produced by MPI cells and by primary alveolar macrophages (AMs) purified from broncho-alveolar lavages of C57BL/6 mice. TNF secretion level was relatively higher in primary AMs infected with *B. pertussis* compared to infected-MPI macrophages even though the difference was not significant (**Figure 5A**). In contrast, MPI cells secreted significantly higher amount of the pro-inflammatory cytokines IL-1β and IL-6 as well as the chemokine MIP-2α (macrophage inflammatory protein 2α) involved in the recruitment of immune cells, than primary AMs in response to *B. pertussis* infection (**Figures 5B–D**). However, and in contrast to primary AMs (29), infected-MPI cells did not secrete IL-22 (**Figure 5E**). No secretion of IL12p40, IL-5 nor IL-10 was detected after *B. pertussis* infection of MPI or primary AMs (data not shown).

### Pro-inflammatory cytokines are produced by *B. pertussis*-infected MPI macrophages in the absence of GM-CSF-induced STAT5 activation

**Figure 6:**
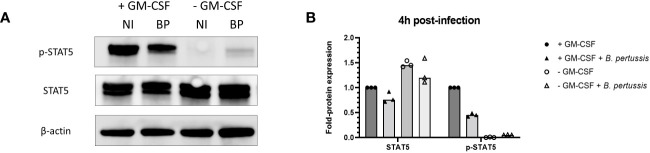
Inhibition of STAT5 phosphorylation in MPI cells cultured without GM-CSF and infected or not with *B. pertussis.* **(A)** Phospho-STAT5 (p-STAT5), STAT5 and β-actin levels detected by immunoblot 4h post-infection in lysates of MPI cells incubated for 1h with *B. pertussis* at a MOI of 50. The end of contact time corresponds to T0. Non-infected cells were added as control. NI: Non-infected cells, BP: *B. pertussis* infected-cells. **(B)** Fold-protein expression of STAT5 and p-STAT5 in *B. pertussis*-infected MPI cells cultured in the presence or not of GM-CSF. Bars represent the median of 3 independent experiments.

**Figure 7:**
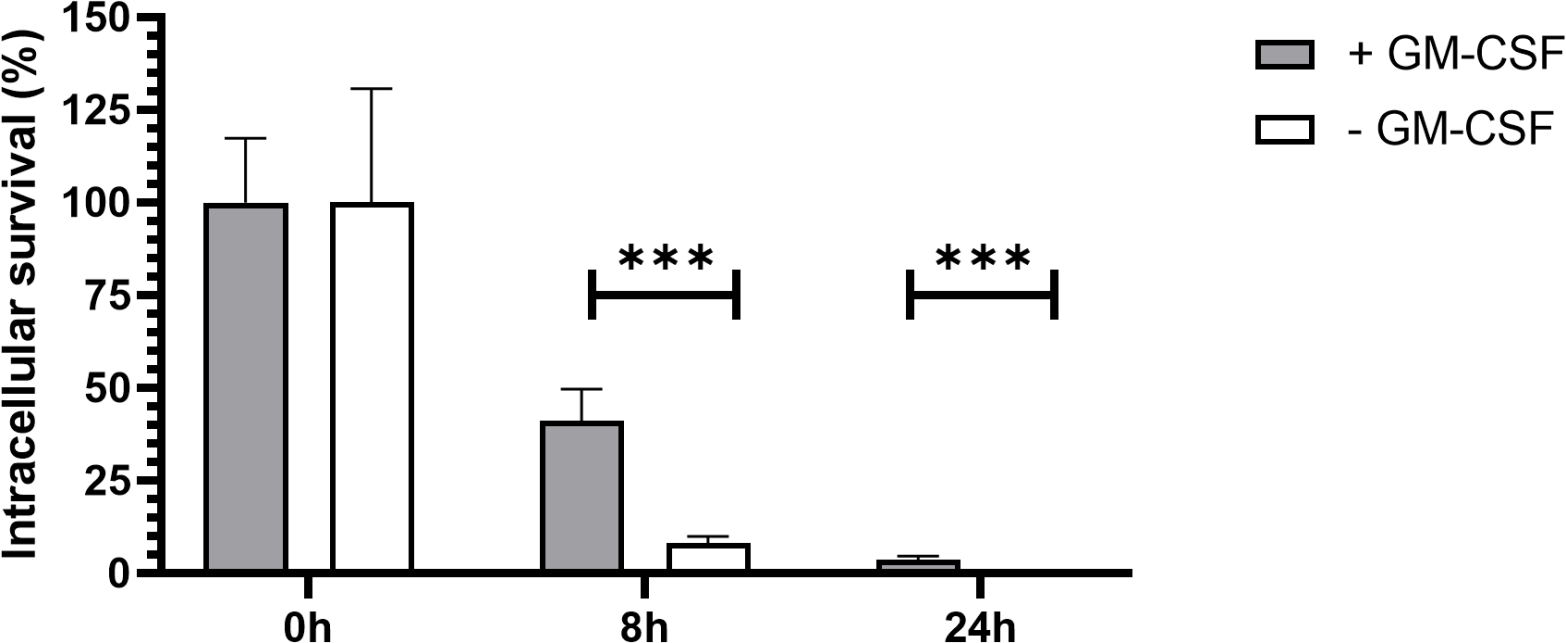
*B. pertussis* clearance in MPI cells with and without GM-CSF. Kinetics of intracellular survival of *B. pertussis* inside MPI cells. MPI cells were incubated for 1h with *B. pertussis* at a MOI of 50. The end of contact time corresponds to T0. Bars represent the mean of quadruplicates with the standard deviation (SD). Data are representatives of 3 independent experiments. ***: p-value <0,001.

**Figure 8:**
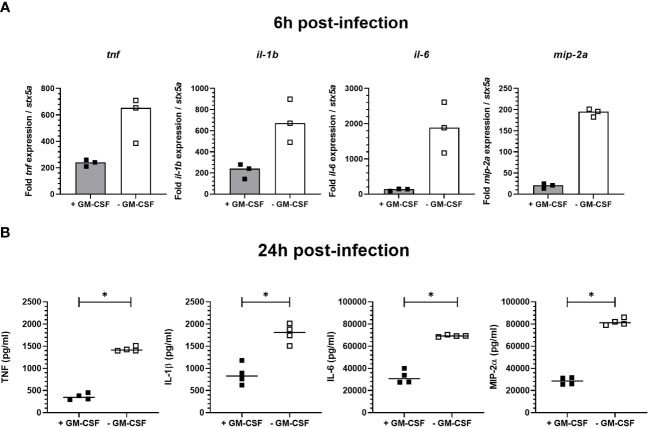
Cytokine expression and secretion by *B. pertussis*-infected MPI cells. MPI cells were incubated for 1h with *B. pertussis* at a MOI of 50. The end of contact time corresponds to T0. **(A)** Expression of *tnf*, *il-1b*, *il-6* and *mip-2a* transcripts in MPI cells cultured in medium containing or not GM-CSF, 6h post-infection. Bars represent the median of triplicates **(B)** Cytokines production measured by ELISA in the supernatants of *B. pertussis*-infected cells 24h post-infection. Lines represent the median of quadruplicates. Statistical test used: Mann-Whitney. Data are representatives of 3 independent experiments. *: p-value < 0,05.

Regulation of signal transduction activator of transcription (STAT) protein family is critical for the induction of pro- or anti-inflammatory responses (30). Among the STAT family, STAT5 was shown to be activated by GM-CSF and to be essential for MPI pro-inflammatory phenotype (19). We therefore assessed the role of STAT5 in the induction of pro-inflammatory cytokine secretion in response to *B. pertussis* infection. Unexpectedly, we found that STAT5 activation, measured by its phosphorylation level, in MPI macrophages is downregulated after *B. pertussis* infection (**Figures 6A, B**). In order to confirm that the production of pro-inflammatory cytokines is independent of GM-CSF-induced STAT5 activation, we cultured MPI cells in medium depleted of GM-CSF before *B. pertussis* infection. As expected, removal of GM-CSF from MPI cells medium resulted in the absence of activation of STAT-5 in uninfected and *B. pertussis*-infected MPI lysates (**Figures 6A, B**). GM-CSF starvation being known to induce G0/G1 cell cycle arrest which could impact cellular properties and lead to comparison biases, we ensured that *B. pertussis* infection was able to induce cell cycle progression despite the removal of GM-CSF from culture medium. Facs analysis of MPI cells cultured in GM-CSF-depleted or complete medium showed that *B. pertussis* infection induced re-entry into the cell cycle as highlighted by an increased number of MPI-infected cells in G2/M phases in both conditions 24h after infection (**Table 1**; **Figure S2**). Moreover, *B. pertussis* killing inside infected-MPI cells cultured with GM-CSF was significantly less efficient than in MPI macrophages grown in a medium without GM-CSF (**Figure 7**). Cellular activity of infected-MPI cells was not affected by the presence or not of GM-CSF (**Figure S3**). Finally, MPI cell phenotype analysed by FACS after 24 hours of GM-CSF starvation showed lower level of CD11c and CD32 compared to MPI cells cultured in medium with GM-CSF while no difference was observed for the other markers studied (SiglecF, CD11b, F4/80, Ly6G) (**Figure S4**). The levels of expression and secretion of cytokines were then measured in *B. pertussis*-infected MPI cells cultured with or without GM-CSF. We showed that in response to *B. pertussis*, higher transcript levels of TNF, IL-1β, IL-6 and MIP-2α were detected 6 hours after infection when MPI were cultured without GM-CSF and therefore when STAT5 is inactivated (**Figure 8A**). In addition, 24h after *B. pertussis* infection, significantly higher levels of TNF, IL-1β, IL-6 and MIP-2α were secreted in supernatants of MPI cells cultured without GM-CSF (**Figure 8B**).

**Table 1:** MPI cell cycle progression to G2 phase following *B. pertussis* infection.

	% of cells in SubGl	% of cells in Gl	% of cells in S	% of cells in G2/M
+ GM-CSF	1,9 +/- 0,5	44 +/- 2,8	27,2 +/- 3,7	24,7 +/- 3,7
+ GM-CSF + *B. pertussis*	3,7 +/- 0,5	43,7 +/- 2,7	15,2 +/- 4,0	37,0 +/- 6,0
- GM-CSF	25,5 +/- 2,3	56,2 +/-1,4	8,3 +/- 0,7	8,6 +/-1,7
- GM-CSF + *B. pertussis*	15,9 +/- 1,9	61,0 +/- 2,7	7,6 +/- 2,3	14,5 +/- 0,8

MPI cells were incubated for 1h with *B. pertussis* at a MOI of 50. The table shows the percentage of MPI cells in the different phases of the cell cycle analysed by Facs 24h post-infection. Experiments was done twice, in triplicates. The results represent the mean of the 6 samples with standard deviation (SD).

### Combined downregulation of STAT5 and Tyr705 STAT3 phosphorylation, but not Ser727 STAT3, by *B. pertussis* infection is associated with an increased pro-inflammatory cytokine production by MPI macrophages

**Figure 9:**
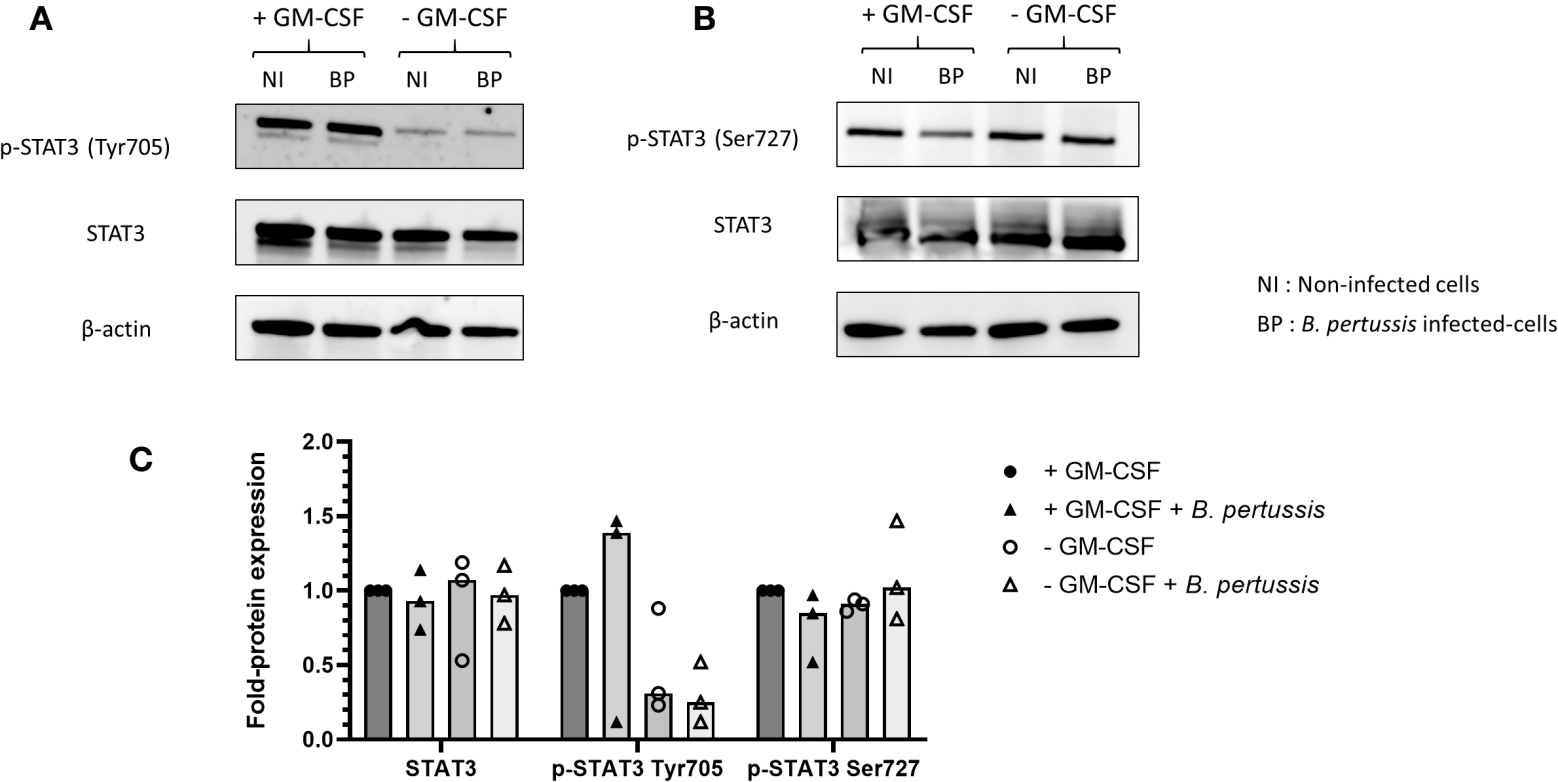
Inhibition of Tyr705 STAT3 phosphorylation, but not Ser727 STAT3, in MPI cells cultured without GM-CSF and infected or not with *B. pertussis.* MPI cells were incubated for 1h with *B.pertussis* at a MOI of 50. The end of contact time corresponds to T0. **(A, B)** Immunoblot of MPI cell lysates infected with *B. pertussis* 4h post-infection, using specific antibodies to detect **(A)** p-STAT3 (Tyr705), **(B)** p-STAT3 (Ser727), **(A, B)** STAT3 and β-actin. NI, Non-infected cells; BP, *B. pertussis* infected-cells. **(C)** Fold-protein expression of p-STAT3 (Tyr705), p-STAT3 (Ser727), STAT3. Results represent the median of 3 independent experiments.

We hypothesized that the surprisingly higher pro-inflammatory cytokines production observed in the absence of GM-CSF-induced STAT5 activation might be linked to the parallel downregulation of STAT3. Indeed, it was shown in a model of intestinal myeloid cells and upon *Salmonella typhimurium* exposure that combined STAT3 and STAT5 inhibition resulted in high level of pro-inflammatory cytokine production (31). We therefore analysed STAT3 activation in *B. pertussis*-infected MPI cells cultured in medium without GM-CSF, conditions where we showed that STAT5 activation was inhibited. We found that similarly to STAT5, activation of STAT3 measured by the phosphorylation of Tyr705 was downregulated compared to *B. pertussis*-infected MPI cells cultured in complete medium (**Figures 9A, C**). STAT3 phosphorylation on Tyr705 induces its translocation to the nucleus to regulate transcription, while Ser727 phosphorylated STAT3 leads to mitochondrial metabolic reprogramming (32). Removal of GM-CSF from MPI cells medium resulted in the downregulation of Tyr705 but not Ser727 STAT-3 phosphorylation after *B. pertussis* infection (**Figures 9B, C**).

## Discussion

Alveolar macrophages are among the first innate cells to encounter *B. pertussis* when the bacteria reach the lungs. Their dual role as a potential reservoir of *B. pertussis* and as a key player in induction of inflammatory and immune responses make them a particularly attractive type of cells to study *B. pertussis* pathogenesis. Our current understanding of the molecular mechanisms induced in AMs in response to *B. pertussis* infection is nevertheless limited due to the constraints in collecting primary AMs which are present in low numbers in the lungs of naïve individuals. Moreover, in the context of 3Rs rules, the use of an alternative cellular model avoiding animals sacrifice is particularly recommended. In this paper, we describe the first data obtained with MPI cells, macrophages phenotypically similar to murine alveolar macrophages (19), in the context of *B. pertussis* infection. We showed in an optimized infection protocol that MPI cells can phagocytose and kill intracellular *B. pertussis.* We observed by transmission electron microscopy that phagosomes containing *B. pertussis* fused with lysosomes, which is a major driver of bacterial killing (33). This is consistent with the decrease in the number of viable intracellular bacteria in MPI cells within 24 hours of infection and is in agreement with a previous study showing colocalization of *B. pertussis* with late endosomal/lysosomal marker LAMP-1 and with Lysotracker, an acidotropic dye, indicating a lysosomal fusion in macrophages derived from blood mononuclear cells (34). In contrast to this study, we haven’t detected multiplication of *B. pertussis* inside MPI macrophages and only very few live bacteria were recovered from infected MPI after 3 days of infection. Macrophages represent a very heterogenous and specialized group of cells (35) and the difference observed between the two studies could be related to the distinct features of AMs compared to monocyte- or bone marrow-derived macrophages (36, 37). Interestingly, a recent study suggested that *B. pertussis* may survive in a dormant, non-culturable state, for a longer time inside macrophages (38). This observation opens a new axis that will have to be further explored in order to understand the intracellular fate of *B. pertussis* inside alveolar macrophages, which have been described as potential infectious reservoir, in particular because of their long lifespan (39).

Another characteristic of alveolar macrophages is that they play a central role in the orchestration of the innate immune response during respiratory infection. In response to *B. pertussis* infection, both MPI macrophages and primary AMs purified from broncho-alveolar lavages of mice exhibit pro-inflammatory phenotypes with the secretion of TNF, IL-1β and IL-6. In addition, we showed that both MPI and primary AMs expressed MIP-2α, also called CXCL2 (40), involved in the recruitment of neutrophils which play a key role in the clearance of *B. pertussis* (41, 42). IL-22 has also been shown to play an important role in mucosal response to gram-negative pathogens, as well as reducing lung injury by promoting mucosal barrier integrity (43). In addition, IL-22 was shown to synergize with IL-17, a cytokine which plays a key role in protective immunity against *B. pertussis* infection (44), to enhance antimicrobial peptide production and neutrophil recruitment (45). We showed here that primary AMs produce IL-22 in response to *B. pertussis* infection and may therefore contribute to the host defense response against *B. pertussis*. MPI cells were previously shown to be unable to produce IL-10 in response to various stimuli (19). Similarly, we did not detect IL-10 production in response to *B. pertussis* infection of MPI macrophages nor IL-22 which belongs to the same family of cytokines as IL-10 (46).

Regulation of cytokine production is a complex pathway, which has been surprisingly poorly studied during *B. pertussis* pathogenesis. In this work, we explored for the first time the role of the signal transduction activator of transcription (STAT) protein family in the innate immune response to *B. pertussis* infection. STAT proteins are part of the Janus kinase-signal transducer and activator of transcription (JAK-STAT) pathway, which is an evolutionary-conserved signalling pathway essential for the regulation of the innate immune response (47). Briefly, the JAKs are activated by binding of extracellular ligands, such as cytokines or growth factors, to transmembrane receptors. Activated JAKs then phosphorylate STAT proteins, which are translocated to the cell nucleus where they will bind to DNA and activate transcription of specific genes (47). Anti-inflammatory cytokines including IL-10 and IL-22 can activate STAT3 and STAT5 in an autocrine/paracrine manner and suppress pro-inflammatory cytokines in response to microbial stimulation in monocytes-derived macrophages (31). Reduced levels of pro-inflammatory cytokines produced by *B. pertussis*-infected primary AMs compared to infected MPI cells might thus be linked to the defect of IL-10 and IL-22 production by MPI macrophages. In addition, among the 7 members of the STAT family, STAT5 phosphorylation is dependent upon GM-CSF stimulation of cells and JAK2 activation (48). GM-CSF is critical for lung alveolar macrophage differentiation, and its presence in the culture medium is required for the proliferation of MPI cells (19, 49). Surprisingly and contrary to a previous study using various stimuli (19), we showed here that in the absence of GM-CSF in the culture medium, high levels of pro-inflammatory cytokines were induced by *B. pertussis* infection of MPI cells suggesting that the induction of pro-inflammatory cytokines by *B. pertussis* is independent of STAT5 activation. In neutrophils, GM-CSF was shown to induce the specific phosphorylation of STAT5 but also STAT3 (50).

Interestingly, STAT3 has been shown to cooperate with STAT5 to regulate the balance between pro- and anti-inflammatory cytokines response and particularly the parallel downregulation of STAT3 and STAT5 resulted in high level of pro-inflammatory cytokine production (31). Similarly, we showed that STAT5 and also STAT3 phosphorylation on Tyr705 was inhibited in MPI cells cultured in medium depleted in GM-CSF and infected with *B. pertussis* and that this combined inhibition was associated with an increase in production of pro-inflammatory cytokines.

GM-CSF specifically leads to tyrosine phosphorylation of JAK2 ( (50, 51) which activates, in addition to STAT5 and STAT3, other major signaling pathways such as PI3K/Akt and Ras/Raf-MAPK/ERK (52). The role of these signalling pathways in modulating the response of alveolar macrophages to *B. pertussis* infection needs to be further explored. In particular, the study of the PI3K/Akt/mTor pathway, involved in cell proliferation, macrophage polarization and in autophagy regulation (53, 54), could provide relevant data to decipher the intracellular survival mechanisms of *B. pertussis* inside alveolar macrophages.

In summary, our study described for the first time the use of a valuable model, called MPI cells, to study the interaction between *B. pertussis* and alveolar macrophages, sentinel cells of the lungs playing a key role in innate immune defense against infection. Future research using *in vitro* 2D or 3D platforms to mimic pulmonary environment will be necessary to further evaluate the role of the cross-talk between AMs and lung epithelial cells (55) in the induction of innate immunity in response to *B. pertussis* infections.

Using this *in vitro* model, we explored for the first time the role of two proteins from the STAT family in the regulation of pro-inflammatory cytokine production induced by *B. pertussis* infection of alveolar macrophages. Our study showed that induction of pro-inflammatory cytokines by *B. pertussis*-infected MPI cells was linked to the combined inhibition of STAT5 and STAT3 activation in the model of MPI alveolar macrophages. While further studies need to be done, this work paves the way toward a better understanding of the role of AMs in innate immunity and *B. pertussis* pathogenesis using MPI as a model to optimize experiments and obtain preliminary data while reducing the number of animals to be sacrificed.

## Data availability statement

The raw data supporting the conclusions of this article will be made available by the authors, without undue reservation.

## Ethics statement

The animal study was approved by Animal experiments were carried out according to the guidelines of the French Ministry of Research on animal experiments and with institutional regulations and ethical guidelines (B59-350009, Institut Pasteur de Lille, France). The protocols were approved by the Ethical Committees of the Region Nord-Pas-de-Calais and the Ministry of Research (agreement number APAFIS # 201603311654342_v2). The study was conducted in accordance with the local legislation and institutional requirements.

## Author contributions

FK: Conceptualization, Data curation, Formal Analysis, Methodology, Validation, Writing – original draft, Writing – review & editing. ZK: Conceptualization, Data curation, Formal Analysis, Investigation, Methodology, Validation, Writing – review & editing. VD: Data curation, Formal Analysis, Investigation, Methodology, Validation, Writing – review & editing. SS: Data curation, Formal Analysis, Investigation, Methodology, Writing – review & editing. EP: Investigation, Methodology, Writing – review & editing. A-SD: Investigation, Methodology, Validation, Writing – review & editing. SC: Data curation, Methodology, Validation, Writing – review & editing. NB: Investigation, Writing – review & editing. CR: Conceptualization, Data curation, Formal Analysis, Investigation, Methodology, Supervision, Validation, Writing – review & editing. NM: Conceptualization, Data curation, Formal Analysis, Funding acquisition, Methodology, Supervision, Validation, Writing – original draft, Writing – review & editing.

## Supplementary material

Supplementary Figure 1 Representative flow cytometry plots showing gating for BAL cells from 8-week old naive mice. Alveolar macrophages were designated as SiglecF + CD11b-.

Supplementary Figure 2 Characterisation of MPI cell cycle in FACS after GM-CSF starvation. Cell cycle analysis of MPI cells cultivated in the presence **(A, B)** of GM-CSF or after 24h starvation **(C, D)** by FACS. A and C show three-dimensional flow cytometry of propidium iodide stained cells. B and D show the relative proportion of cells in each phase of the cycle and was calculated from A and C with the Dean-Jett-Fox model using FlowJo software. Experiment was repeated at least three times, and the data shown here are representative of the results obtained.

Supplementary Figure 3 MPI cell activity cultured with or without GM-CSF and infected with *B. pertussis*. MPI cell activity assessed using MTT assay, 24h after infection of MPI cells with *B. pertussis* at a MOI of 50 for 1h. Statistical test used: Kruskal-Wallis followed by a Dunn’s test for multiple comparisons. Lines represent the median of five biological replicates and the graph is representative of 2 experiments. ***: p-value <0,001.

Supplementary Figure 4 Characterisation of MPI cell phenotype by FACS after GM-CSF starvation. Representative flow cytometry charts of SiglecF, CD11b, CD11c, CD32, F4/80, Ly6G expression by MPI cells cultured in presence of GM-CSF or after 24h of GM-CSF starvation. Data shown here are representative of the results obtained for 10 replicates.

Supplementary Table 1 Primers for the genes of interest analyzed by qRT-PCR. SP: Sense primer; ASP: Anti-Sense primer.
