# Supplementary figures and images for: Combined regulation of pro-inflammatory cytokines production by STAT3 and STAT5 in a model of B. pertussis infection of alveolar macrophages
Source: Front Immunol. 2023 Sep 28;14:1254276. doi: 10.3389/fimmu.2023.1254276 (PMC10569487; doi:10.3389/fimmu.2023.1254276)

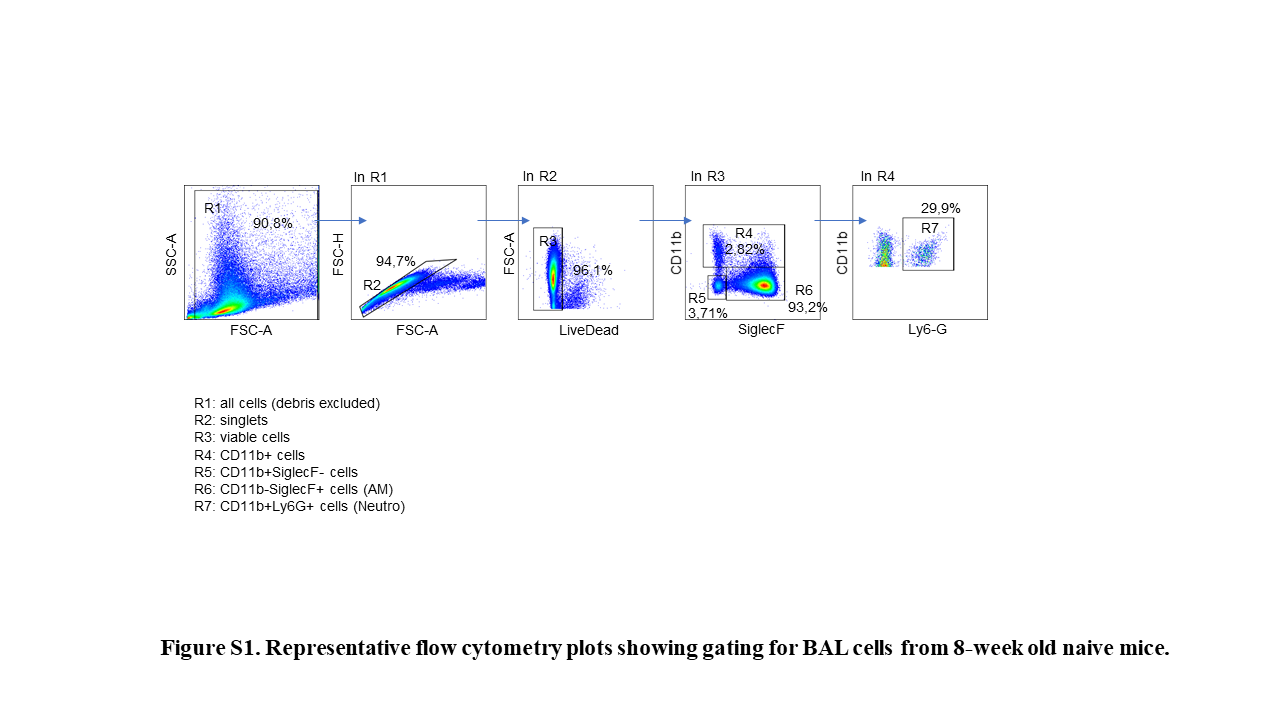

Supplement: Supplementary Figure 1 — Representative flow cytometry plots showing gating for BAL cells from 8-week old naive mice. Alveolar macrophages were designated as SiglecF + CD11b-. [file Image_1.tif]

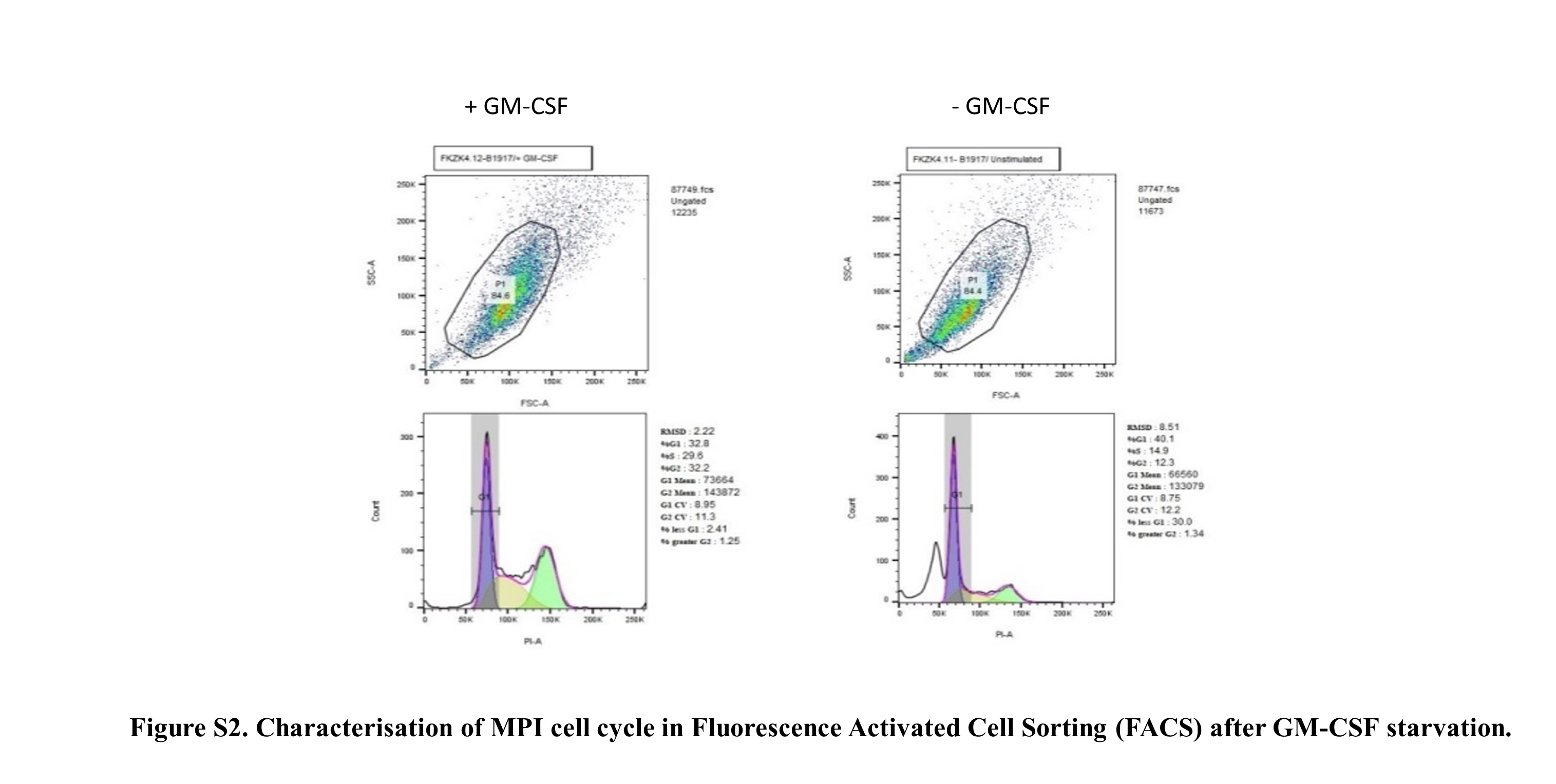

Supplement: Supplementary Figure 2 — Characterisation of MPI cell cycle in FACS after GM-CSF starvation. Cell cycle analysis of MPI cells cultivated in the presence (A, B) of GM-CSF or after 24h starvation (C, D) by FACS. A and C show three-dimensional flow cytometry of propidium iodide stained cells. B and D show the relative proportion of cells in each phase of the cycle and was calculated from A and C with the Dean-Jett-Fox model using FlowJo software. Experiment was repeated at least three times, and the data shown here are representative of the results obtained. [file Image_2.tif]

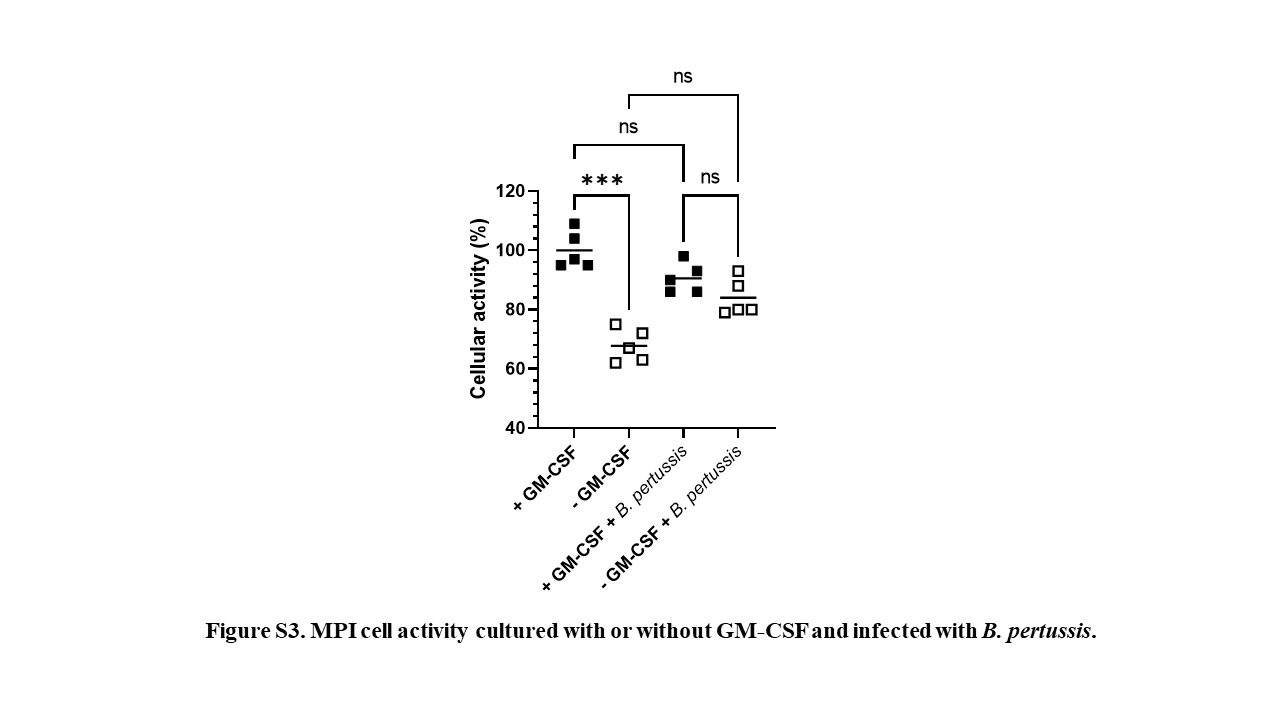

Supplement: Supplementary Figure 3 — MPI cell activity cultured with or without GM-CSF and infected with B. pertussis. MPI cell activity assessed using MTT assay, 24h after infection of MPI cells with B. pertussis at a MOI of 50 for 1h. Statistical test used: Kruskal-Wallis followed by a Dunn’s test for multiple comparisons. Lines represent the median of five biological replicates and the graph is representative of 2 experiments. ***: p-value <0,001. [file Image_3.tif]

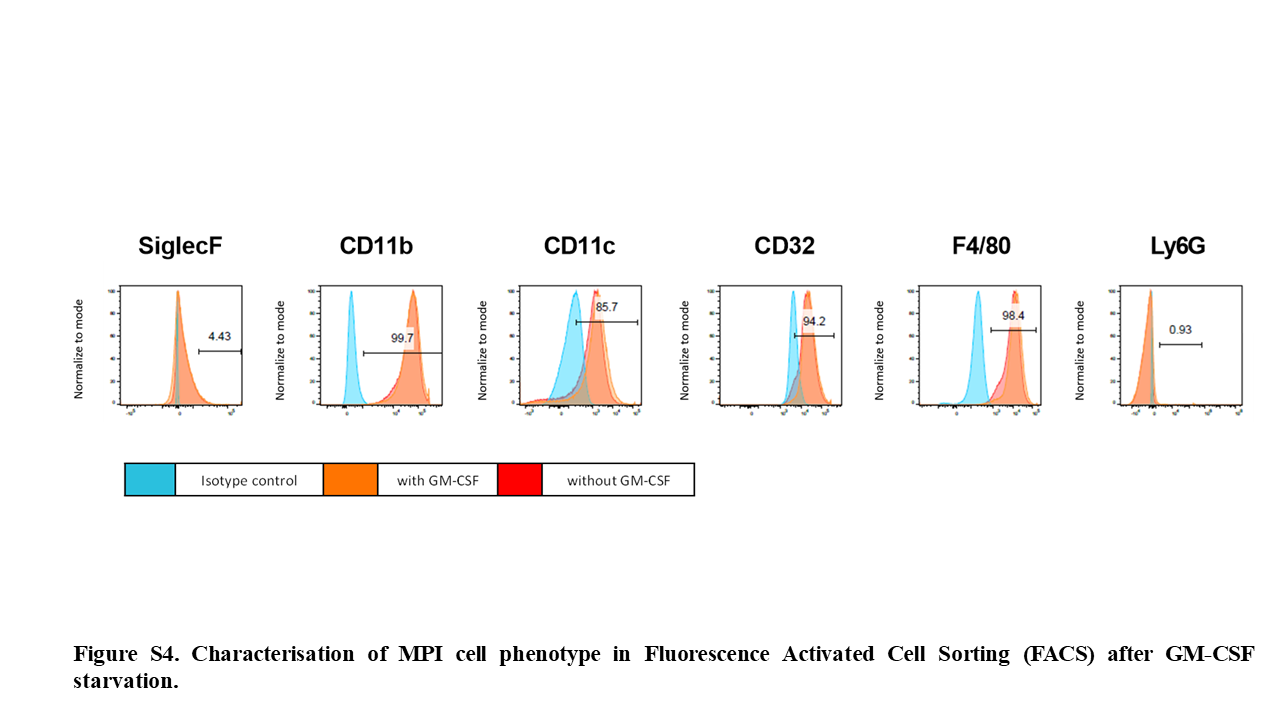

Supplement: Supplementary Figure 4 — Characterisation of MPI cell phenotype by FACS after GM-CSF starvation. Representative flow cytometry charts of SiglecF, CD11b, CD11c, CD32, F4/80, Ly6G expression by MPI cells cultured in presence of GM-CSF or after 24h of GM-CSF starvation. Data shown here are representative of the results obtained for 10 replicates. [file Image_4.tif]

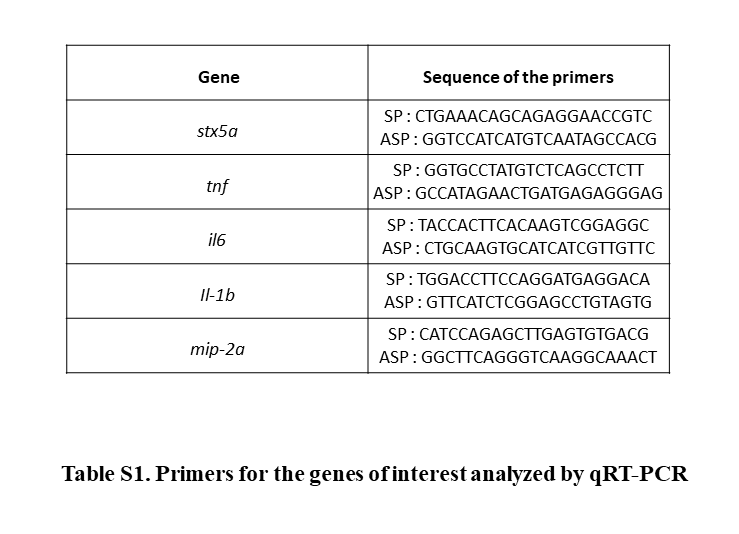

Supplement: Supplementary Table 1 — Primers for the genes of interest analyzed by qRT-PCR. SP: Sense primer; ASP: Anti-Sense primer. [file Table_1.docx]
